# The clinic-based predictive modeling for prognosis of patients with cryptococcal meningitis

**DOI:** 10.1186/s12879-023-08337-2

**Published:** 2023-05-25

**Authors:** Chen Zhang, Zixian He, Zheren Tan, Fafa Tian

**Affiliations:** 1 Departments of Neurology, Xiangya Hospital, Central South University, 87 Xiangya Road, Changsha, 410008 China; 2 Biomedial Engineering major, College of Engineering, Boston University, 25 Buick street, Boston, MA 02215 USA; 3 Departments of Critical Care Medicine, Xiangya Hospital, Central South University, 87 Xiangya Road, Changsha, 410008 China

**Keywords:** Cryptococcal meningitis, Receiver-operating characteristic curve, Prognosis prediction, Immunocompetent patients

## Abstract

**Background:**

Cryptococcal meningitis (CM) is the most common fungal infection of the central nervous system that can cause significant morbidity and mortality. Although several prognostic factors have been identified, their clinical efficacy and use in combination to predict outcomes in immunocompetent patients with CM are not clear. Therefore, we aimed to determine the utility of those prognostic factors alone or in combination in predicting outcomes of immunocompetent patients with CM.

**Methods:**

The demographic and clinical data of patients with CM were collected and analyzed. The clinical outcome was graded by the Glasgow outcome scale (GOS) at discharge, and patients were divided into good (score of 5) and unfavorable (score of 1–4) outcome groups. Prognostic model was created and receiver-operating characteristic curve analyses were conducted.

**Results:**

A total of 156 patients were included in our study. Patients with higher age at onset (p = 0.021), ventriculoperitoneal shunt placement (p = 0.010), Glasgow Coma Scale (GCS) score of less than 15(p< 0.001), lower CSF glucose concentration (p = 0.037) and immunocompromised condition (p = 0.002) tended to have worse outcomes. Logistic regression analysis was used to create a combined score which had a higher AUC (0.815) than those factors used alone for predicting outcome.

**Conclusions:**

Our study shows that a prediction model based on clinical characteristics had satisfactory accuracy in prognostic prediction. Early recognition of CM patients at risk of poor prognosis using this model would be helpful in providing timely management and therapy to improve outcomes and to identify individuals who warrant early follow-up and intervention.

**Supplementary Information:**

The online version contains supplementary material available at 10.1186/s12879-023-08337-2.

## Background

Cryptococcal meningitis (CM) is the most common fungal infection of the central nervous system (CNS) [1]. Despite advances in new anti-fungal agents, CM remains associated with a high morbidity and mortality among immunocompromised patients, such as HIV-infected patients, patients with hematological malignancies, solid-organ transplant recipients and so on [2–4]. Studies have shown no differences in hospital mortality or satisfactory outcomes between immunocompromised and immunocompetent patients [5]. However, the vast majority of research on identifying prognostic has factors of CM focused on immunocompromised patients, such as HIV-positive population, rather than immunocompetent patients. In our previous retrospective study, we found impaired consciousness and decreased glucose concentration in cerebrospinal fluid (CSF) were independent prognostic factors that predict the unsatisfactory outcome in immunocompetent patients with CM [6]. Although the clinical efficacy of combining of multiple prognostic factors in CM is not well investigated, our findings highlight the need to develop practical tools for early recognition of CM patients at risk of poor prognosis. This can facilitate timely management and therapy to improve outcome and identify individuals who warrant early follow-up and intervention.

In this study, we aimed to explore the clinical significance of those prognostic clinical signatures used alone or in combination in the prognostic prediction of patients with CM.

## Methods

We conducted a review of hospitalized patients with CM from January 2003 to August 2022.The diagnosis of CM was based on clinical features and positive laboratory findings. The patients with CM were included if they had to meet one or more of the following criteria: (1) positive culture of cryptococcus from CSF, (2) positive CSF india ink testing, (3) positive CSF cryptococcal antigen testing, or (4) positive cerebral biopsy. Patients who had one or more identifiable underlying diseases were categorized as immunocompromised hosts, this included individuals with a history of autoimmune disorders, long-term glucocorticoids or other immunosuppressive therapies, idiopathic CD4 T-cell lymphopenia, HIV infection, malignant tumor, hepatic cirrhosis, end-stage renal failure or diabetes [6, 7]. Meanwhile, T-SPOT, tuberculosis ELISA, AFB stain, culture in CSF were performed in all cases to exclude the possibility of tuberculosis.

In this retrospective study, we collected demographic data, major symptoms and signs, neuroimaging features, laboratory findings and clinical outcome at discharge. The clinical outcome was evaluated by a neurological physician using Glasgow Outcome Scale (GOS) within 24 h prior to discharge. Score of 1–4, indicating death, vegetative status, severe disability and moderate disability, was considered “unfavorable” clinical outcomes. Score of 5, indicating no or mild disability, was considered “good” outcome [6]. The degree of impaired consciousness at admission was graded by the Glasgow Coma Scale (GCS), which evaluates motor responsiveness, verbal performance, and eye opening, and GCS score can range from 3 (completely unresponsive) to 15 (responsive) and provide a practical method for reflecting the level of consciousness [8].

Mean and standard deviations were presented for parametric variables, while medians and quartiles were used for non-parametric variables. Chi squared and fisher’s exact tests, Two-sample t-test (parametric) or Mann Whitney U test(non-parametric) were used to assess differences in demographic and clinical features between the good and unfavorable outcome groups. Variable selection was performed using the least absolute shrinkage and selection operator (LASSO) regression model, followed by multivariable logistic regression analysis to create a combined score for predicting the outcome utilizing the independent variable statistically significant at the univariate analysis, in which P value levels for inclusion and exclusion criteria were set as 0.05 and 0.10, respectively. Odds ratio (OR) and its 95% Confidence interval (CI) were estimated for each factor. To determine clinical efficacy of individual variables and the combined predictive score. The pairwise comparison of ROC curves was conducted with Delong’s test. The area under the curve (AUC) with 95% CI that evaluates the sensitivity and specificity, and cut-off values were calculated. P values < 0.05 were considered statistically significant. All statistical analyses in this study were conducted using SPSS (version 23.0, Chicago, IL, USA) and MedCalc Statistical Software (Ostend, Belgium).

## Results

A total of 156 confirmed patients with CM (95 males; range: 16–87 years) with complete data were included, and 49 of them were considered immunocompromised.

In our study, the most frequent symptoms included headache (89.7%), fever (55.8%), vomiting (41.0%). Demographics and clinical manifestations of all cases in the training set are listed in Table 1.

**Table 1:** Demographic and clinical profile of patients with Cryptococcal meningitis

Variable	Value
Gender, M/F	95/61 (60.9%/39.1%)
Age at onset(years)	50.0 (41.3–62.0)
interval from onset to antifungal treatment(day)	31.0(18.0,50.0)
duration of antifungal treatment(day)	21.0(11.0, 40.0)
Am B administration	148 (96.9%)
Shunt surgery	22 (14.1%)
Main symptoms and signs	
Headache	140 (89.7%)
Fever	87 (55.8%)
Vomiting	64 (41.0%)
Impaired consciousness(GCS score<15)	19 (12.2%)
Visual disturbance	21 (13.5%)
Seizures	12 (7.7%)
Limb weakness	14 (9.0%)
Psychiatric symptoms	12 (7.7%)
Hearing impairment	7 (4.5%)
Meningeal irritation positive	62 (39.7%)

Data are expressed as n (%) or median (interquartile range); Am B: amphotericin B;

87.8% of patients (n = 137) had the highest GCS score of 15. The median white blood cell (WBC) count in the blood was 8.2 (interquartile range, IQR 5.6, 12.0) × 10^9^/L. The median WBC count in the CSF was 54.0(IQR 19.3, 149.0) 10^6^/L, the median CSF glucose concentration was 2.18 (IQR 1.16, 2.94) mmol/L. The median CSF chloride concentration was 117.6 (IQR 114.6, 121.4) mmol/L. The median CSF protein concentration was 0.87 (IQR 0.52, 1.54) g/L. The sensitivity of the CSF India ink test/antigen test and culture in our study were 82.1% and 48.1%, respectively. One patient was diagnosed with CM by positive cerebral biopsy. Laboratory data and features of neuroimaging were presented in Table 2. At discharge, we assessed the outcome for all patients by using GOS, 60 patients (38.5%) obtained a good outcome.

**Table 2:** laboratorial findings and Neuroimaging of patients with Cryptococcal meningitis

Variable	Value
Blood WBC count(10^9^/L)	8.2(5.6, 12.0)
CSF	
Opening pressure(> 180mmH2O)	127(81.4%)
WBC count(10^6^/L)	54.0(19.3, 149.0)
Glucose(mmol/L)	2.18(1.16, 2.94)
Chloride (mmol/L)	117.6 (114.6, 121.4)
Protein (g/L)	0.87 (0.52, 1.54)
India ink test or antigen test positive	128(82.1%)
Culture positive	76 (48.7%)
Cerebral biopsy positive	1(0.6%)
Neuroimaging	
Parenchymal lesions	69(44.2%)
Meningeal enhancement	41(26.3%)
Hydrocephalus	19(12.2%)

Data are expressed as n (%) or median (interquartile range); WBC: white blood cell; CSF: cerebrospinal fluid

In a univariate analysis comparing the good outcome group with the unfavorable outcome group, patients with higher age at onset (*p* = 0.021), ventriculoperitoneal shunt placement (*p* = 0.010), GCS score of less than 15(*p*< 0.001), lower CSF glucose concentration (*p* = 0.037) and immunocompromised condition (*p* = 0.002) tended to have worse outcomes (Table 3).

**Table 3:** Results of univariate analysis identifying variables that differed significantly between the good and unfavorable outcome groups

Variable	Good (n = 60)	Unfavorable (n = 96)	Pvalue	Logistic regression		
				B	OR (95% CI)	p value
Gender (male)	34	61	0.392^*^	-0.287	0.750(0.388,1.449)	0.392
Age at onset (years)	46.5(36.3,56.0)	50.0(43.0,64.0)	**0.021**	0.030	1.031(1.007,1.055)	**0.010**
Interval from onset to antifungal treatment(day)	29.0(15.0,39.0)	34.5(18.3,63.8)	0.082	0.007	1.007(0.999,1.014)	0.095
Duration of antifungal treatment(day)	23.0(13.0,44.0)	16.5(8.0,39.0)	0.131	-0.003	0.997(0.987,1.007)	0.554
Am B administration	57	91	> 0.999^*^	-0.043	0.958(0.220,4.163)	0.954
Ventriculoperitoneal shunt placement	3	19	**0.010** ^*^	1.545	4.688(1.323,16.609)	**0.017**
Meningeal irritation positive	18	44	0.098^*^	0.680	1.974(0.998,3.908)	0.051
CSF pressure (> 180mmH2O)	49	78	0.948^*^	0.028	1.028(0.448,2.359)	0.948
Blood WBC count(10^9^/L)	7.8(5.8,10.8)	8.4(5.6,12.3)	0.457	0.028	1.028(0.955,1.108)	0.462
CSF WBC count(10^6^/L)	65.0(20.0,165.8)	50.0(18.0,121.3)	0.412	-0.001	0.999(0.998,1.001)	0.328
CSF Glucose(mmol/L)	2.5(1.7,2.9)	1.8(0.9,2.9)	**0.037**	-0.118	0.889(0.701,1.128)	**0.332**
CSF Chloride (mmol/L)	118.7(115.6,122.3)	117.3(113.6,121.3)	0.342	-0.019	0.981(0.938,1.027)	0.410
CSF Protein (g/L)	0.85(0.53,1.33)	0.93(0.52,1.55)	0.600	0.193	1.213(0.776,1.896)	0.397
Parenchymal lesions	28	41	0.669^*^	-0.142	0.868(0.453,1.662)	0.669
Meningeal enhancement	11	30	0.091^*^	0.705	2.025(0.925,4.433)	0.078
Hydrocephalus	5	14	0.236^*^	0.643	1.901(0.648,5.582)	0.242
GCS score(<15)	1	25	**<0.001** ^*^	3.034	20.775(2.733,157.924)	**0.003**
Immunocompromised condition	10	39	**0.002** ^*^	1.230	3.421(1.550,7.551)	**0.002**

Data are expressed as n (%) or median (interquartile range); Statistical analysis rank sum test unless otherwise specified; *chi-square test; Bold denotes statistical difference; Am B: Amphotericin B; CSF: cerebrospinal fluid; GCS score : Glasgow Coma Scale score; WBC: white blood cell; CSF: cerebrospinal fluid; B:Regression coefficient;OR: Odds ratio; CI: Confidence interval

**Fig. 1:**
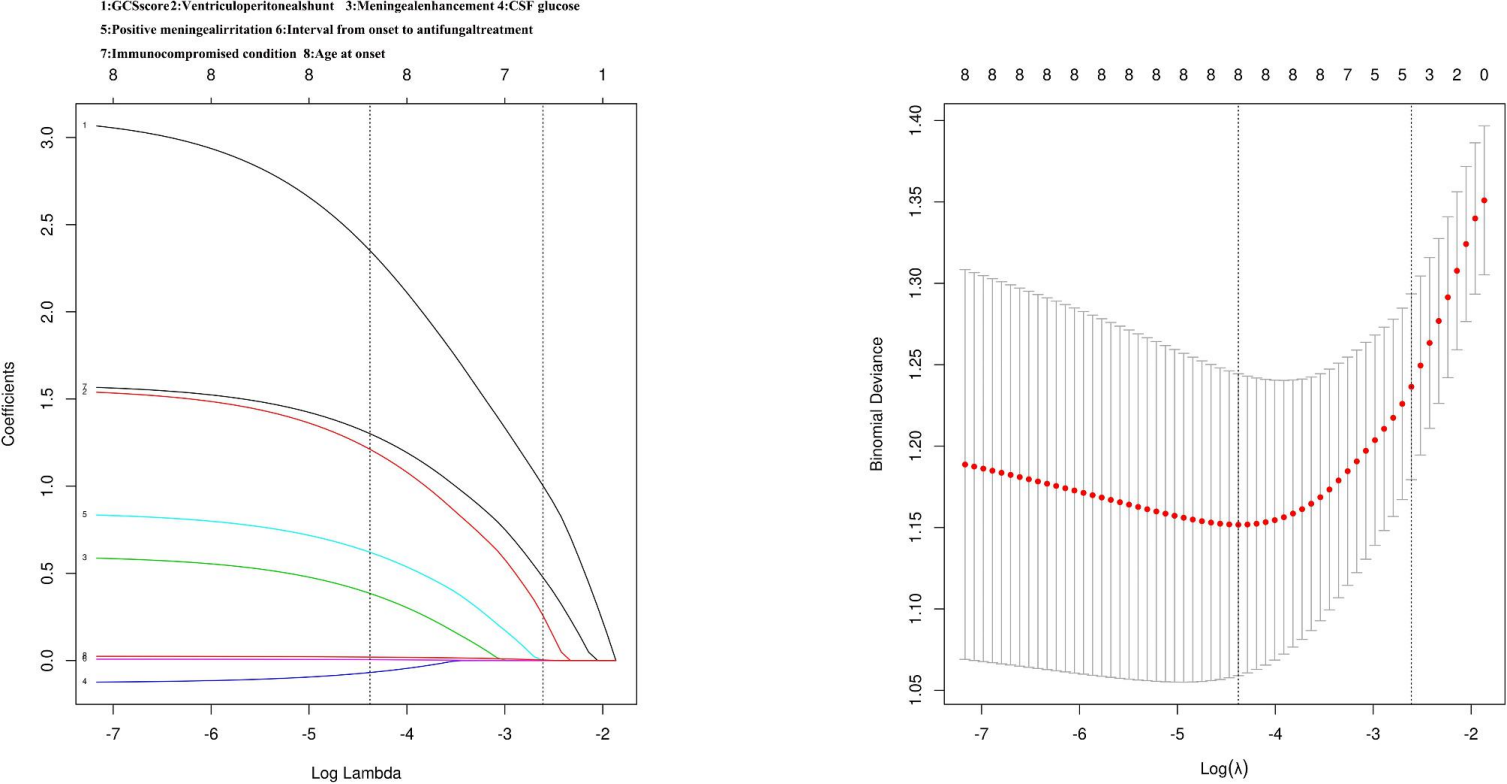
Variables selection using the LASSO logistic regression. LASSO, least absolute shrinkage and selection operator; CSF, cerebrospinal fluid; GCS, glasgow coma score

For variable selection, all variables(n = 8) with p-value of less than 0.1 in univariate analysis were included in LASSO, lambda.min and lambda.1se (standard error, SE) were 0.013 and 0.074. Optimal lambda.min resulted in 8 nonzero coefficients (Fig. 1). To identify the combined score, a multivariable logistic regression analysis was conducted and 8 variables with nonzero coefficients in LASSO were included. The result was the following: combined score = 1.587*(immunocompromised condition:0 for without, 1 for with) + 1.563*( ventriculoperitoneal shunt : 0 for without, 1 for with) + 0.025*(age at onset)-0.128*(CSF glucose concentration) + 3.129*(GCS score: 0 for = 15,1 for<15) + 0.602*(meningeal enhancement:0 for without,1 for with) + 0.851*(positive meningeal irritation: 0 for without, 1 for with) + 0.008*(Interval from onset to antifungal treatment). The results of a multivariable logistic regression were presented in Table 4. Mann Whitney U test confirmed that patients with higher combined score tended to have worse outcomes(p<0.001).

**Table 4:** Results of multiple logistic regression analysis for prognostic model

Variable	B	OR (95% CI)	Pvalue
Age at onset	0.025	1.025(0.998,1.052)	0.068
Interval from onset to antifungal treatment	0.008	1.008(0.998,1.018)	0.102
Ventriculoperitoneal shunt placement	1.563	4.775(1.168,19.524)	0.030
Meningeal irritation positive	0.851	2.342(0.991,5.536)	0.052
CSF Glucose	-0.128	0.880(0.652,1.189)	0.405
Meningeal enhancement	0.602	1.827(0.714,4.674)	0.209
GCS score	3.129	22.844(2.719,191.957)	0.004
Immunocompromised condition	1.587	4.887(1.985,12.031)	0.001
Constant	-2.147		0.017

CSF: Cerebrospinal fluid; B:Regression coefficient;OR: Odds ratio; CI: Confidence interval

**Fig. 2:**
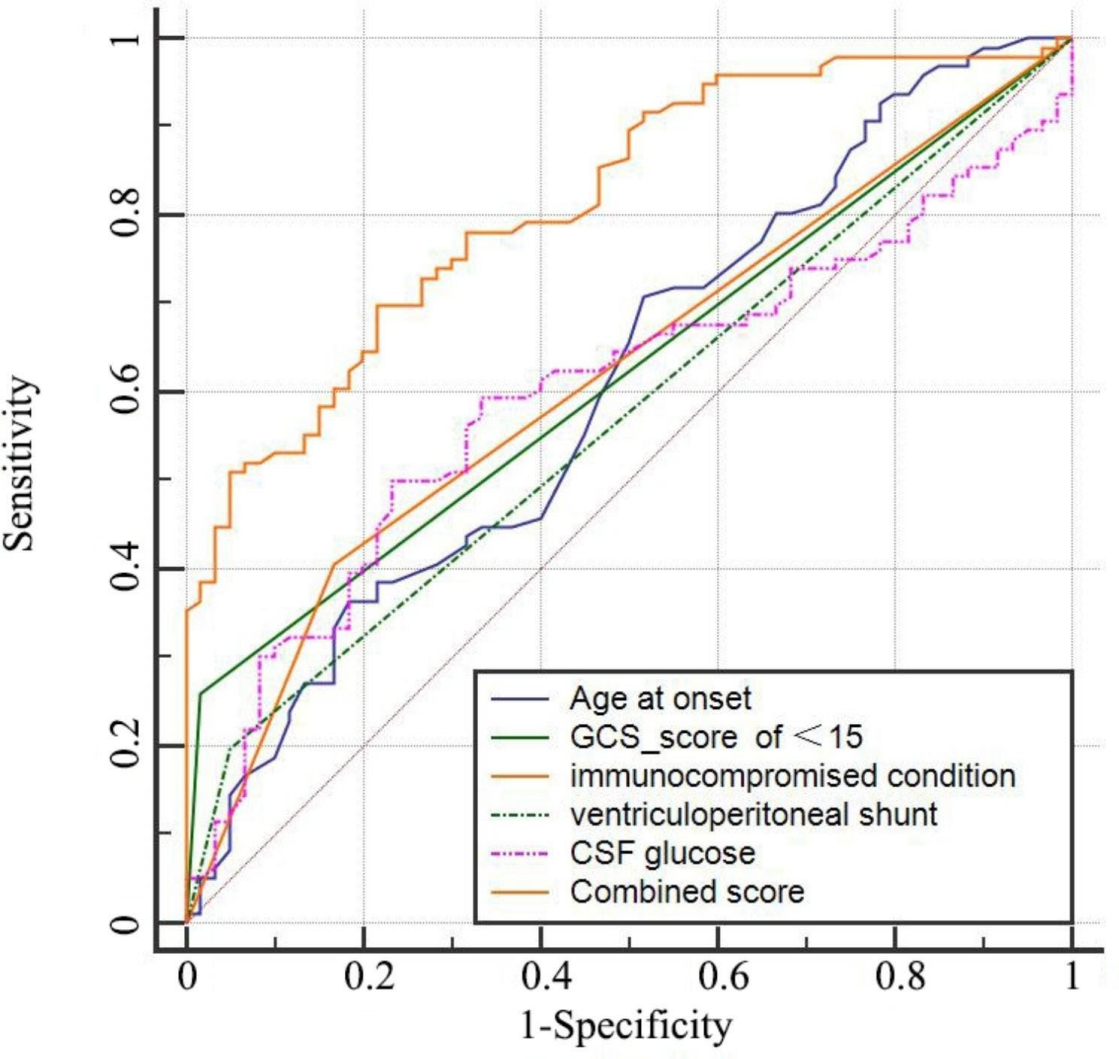
ROC analysis of individual factor and combined score in predicting outcome of patients with CM. ROC, receiveroperating characteristic; CSF, cerebrospinal fluid; GCS, glasgow coma score; CM, cryptococcal meningitis

Receiver operating characteristic (ROC) curve was performed to investigate the predictive value of those markers used alone and combined score. We found that CSF glucose concentration(sensitivity of 50.0%, Specificity of 76.7%, AUC of 0.599), GCS score of <15(sensitivity of 26.0%, Specificity of 98.3%, AUC of 0.622), age at onset (sensitivity of 70.8%, Specificity of 48.3%, AUC of 0.610), ventriculoperitoneal shunt placement (sensitivity of 19.8%, Specificity of 95.0%, AUC of 0.574), immunocompromised condition (sensitivity of 40.6%, Specificity of 83.3%, AUC of 0.620) and combined score(sensitivity of 69.8%, Specificity of 78.3%, AUC of 0.815) had significant accuracy for predicting the unfavorable outcomes in patients with CM (p<0.05 for all, Table 5). According to multiple comparisons, the combined score provided higher AUC compared with the CSF glucose concentration, GCS score of <15, age at onset, immunocompromised condition and ventriculoperitoneal shunt placement (p<0.001 for all, Fig. 2), respectively. The cut‑off values and AUC values are displayed in Table 5.

**Table 5:** Use of cut off values of individual factors and combined score for predicting unfavorable outcome of patients with CM.

Variable	Cut-off value	Pvalue	AUC	Sensitivity,	Specificity,	Accuracy,
CSF glucose	≤ 1.71 mmol/L	0.029	0.599	50.0%	76.7%	60.3%
GCS score	≤ 14	<0.001	0.622	26.0%	98.3%	53.8%
Age at onset	>45 yrs	0.018	0.610	70.8%	48.3%	62.2%
ventriculoperitoneal shunt placement	presented	0.003	0.574	19.8%	95.0%	48.7%
Immunocompromised condition	presented	<0.001	0.620	40.6%	83.3%	57.1%
Combined score	>2.67	<0.001	**0.815**	69.8%	78.3%	**73.1%**

CM, cryptococcal meningitis; CSF: cerebrospinal fluid; AUC: area under the curve

## Discussion

Previous studies have consistently confirmed that high CSF glucose concentration and age at onset are independent prognostic factors associated with favorable prognosis, mortality, survival time, regardless of presence of predisposing diseases [9–14]. Our study supports these findings.

Our study also confirms that ventriculoperitoneal shunt placement is an independent prognostic factor associated with unfavorable prognosis. This surgical procedure is commonly used in the management of CNS infection and is typically performed in patients with severe hydrocephalus or increased intracranial pressure, which lead to extensive brain injury and neurodisability[15].

Our present study identified a GCS score below 15 and immunocompromised conditions as independent prognostic factor. While previous studies have demonstrated that higher GCS score are associated with favorable outcomes [16] and lower mortality [17]. Li et al. found that no association between immunocompromised condition and worse prognosis or mortality in non-HIV population with CM [5]. One possible explanation for this difference is that our study included 5 AIDS patients (10% of the immunocompromised population) and more patients with long-term use of corticosteroid (46.9% vs. 23.2%, n = 23), which indicated that a greater degree of immunosuppression in our study.

In the present study, the AUC and cut-off values of individual and combined score have been analyzed. Compared with factors used alone, the combined score turned out to be a satisfactory predictor with an AUC of 0.815, which indicated that the patients with combined score of >2.67 had a significantly higher probability of unfavorable outcome. Besides that, the combined score was easy to be applied in practice, because these factors could be obtained from the routine examinations and scales without any additional costs in the diagnosis of CNS infections.

The prognostic model developed in the present study may draw the attention of clinicians to provide early specific measures, such as the admission of patients with a higher risk of poor outcome to intensive care units (ICU). Additionally, it could provide a helpful tool for risk assessment and decision-making in treatment strategy. Identifying patients with a higher risk of poor outcome could facilitate earlier, aggressive treatment (e.g., maximum dose and duration of Liposomal AmB in the induction phase) and potentially improve outcomes. Conversely, identifying patients with a lower risk of poor outcome could enable the use of moderate treatment to relieve the side effects and the financial burden caused by long-term antifungal therapy.

There were some limitations on the strength of this study. A larger sample size may allow findings to be more accurate. Moreover, no data of long-term clinical outcome after discharge were recruited, the utility of the clinical factors here analyzed on predicting long term prognosis was unattainable in this study. Because the vast majority of patients have not undergone CrAg test, the finding of the CrAg test was not included in our study. Therefore, a multicenter study with long term follow-up data and more variables should be performed for a more detailed study.

## Conclusions

The present study established a prediction model based on clinical characteristics, which had statistically significant accuracy in prognostic prediction. Our findings may help clinicians early identify patients with poor outcomes and optimize treatment strategy.

## Electronic supplementary material

Below is the link to the electronic supplementary material.

Supplementary Material 1

## Data Availability

The data that support the findings of this study are available from the corresponding author via E-mail upon reasonable request.
