# Supplementary material for: The clinic-based predictive modeling for prognosis of patients with cryptococcal meningitis
Source: BMC Infect Dis. 2023 May 25;23:352. doi: 10.1186/s12879-023-08337-2 (PMC10210386; doi:10.1186/s12879-023-08337-2)

supplementary figure1：ROC analysis of predictive model based on different parameters in predicting outcome of patients with CM. the LASSO was conducted for variable selection. Blue curve represent the predictive model in which 8 variables are included based on lambda.min,the AUC of blue curve is 0.815;Green curve represent the predictive model in which just 4 variables are included based on lambda.1se,the AUC of green curve is 0.781. ROC, receiver‑operating characteristic; CM, cryptococcal meningitis.


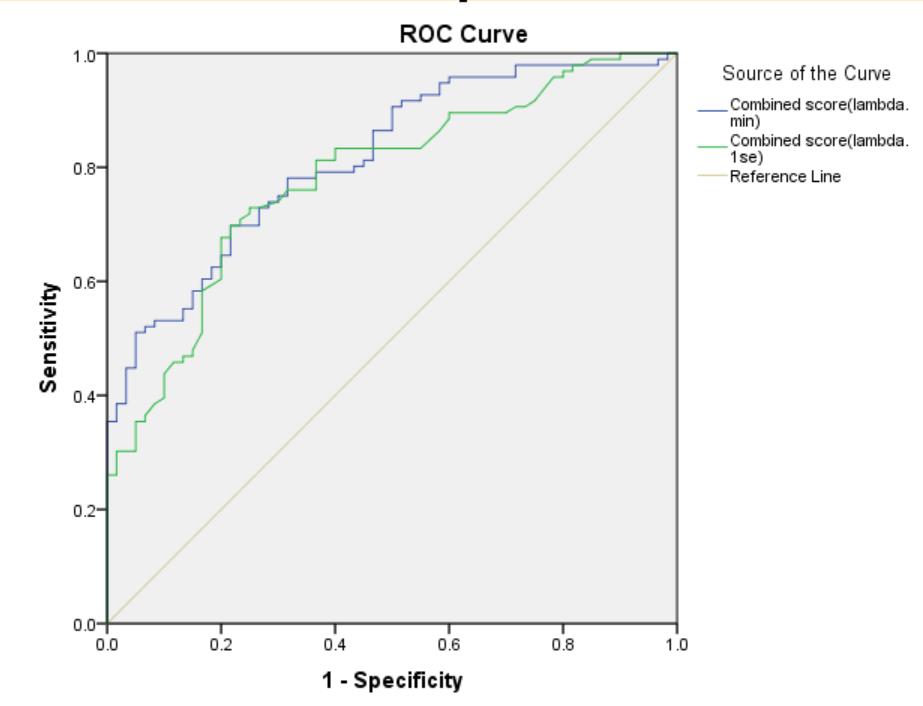

Supplement: Supplementary file 1 — Supplementary Material 1 [file 12879_2023_8337_MOESM1_ESM.docx]
